# Metal Homeostasis in Pathogenic Streptococci

**DOI:** 10.3390/microorganisms10081501

**Published:** 2022-07-25

**Authors:** Madeline S. Akbari, Kelly S. Doran, Lindsey R. Burcham

**Affiliations:** Department of Immunology and Microbiology, School of Medicine, University of Colorado Anschutz Medical Campus, Aurora, CO 80045, USA; madeline.akbari@cuanschutz.edu

**Keywords:** Streptococci, metal homeostasis, metal transport, virulence, zinc, manganese, iron, nickel, copper

## Abstract

*Streptococcus* spp. are an important genus of Gram-positive bacteria, many of which are opportunistic pathogens that are capable of causing invasive disease in a wide range of populations. Metals, especially transition metal ions, are an essential nutrient for all organisms. Therefore, to survive across dynamic host environments, Streptococci have evolved complex systems to withstand metal stress and maintain metal homeostasis, especially during colonization and infection. There are many different types of transport systems that are used by bacteria to import or export metals that can be highly specific or promiscuous. Focusing on the most well studied transition metals of zinc, manganese, iron, nickel, and copper, this review aims to summarize the current knowledge of metal homeostasis in pathogenic Streptococci, and their role in virulence.

## 1. Introduction

Metalloproteins require metal ions to function properly, and it is suggested that almost half of all known proteins require at least one metal ion cofactor [1]. For this reason, metals are necessary for the survival of all organisms, as they play direct roles in growth, replication, and cellular metabolism [1,2]. The homeostasis of metal ions, especially of divalent cations, is therefore a necessary and tightly regulated process that is essential for survival. Conditions of metal ion imbalance can result in the mismetallation of metalloproteins or the production of reactive oxygen species (ROS) through the Fenton reaction, both of which can be toxic to cells [3]. Metal deplete and replete environments can have dramatic and detrimental effects on organisms if they are unable to respond to metal stress or do not have mechanisms to maintain optimal intracellular metal concentrations [4,5]. Bacterial metal transport systems are key players in maintaining metal homeostasis, and the transport systems in *Streptococcus* spp. have begun to be elucidated.

Metal transport systems are conserved throughout bacterial species [6], and one of the largest superfamilies of proteins and most common transporter types are the ATP-binding cassette transporters (ABC transporters) [7]. ABC transporters typically consist of a substrate binding protein (SBP), one or two hydrophobic membrane-spanning permeases, and one or two nucleotide-binding ATPases that supply the energy for active transport of the metal ion. SBPs are lipoproteins that localize to the membrane via the secretory (Sec) pathway in Gram-positive bacteria and are further organized into eight clusters, named A–H, based on protein structure and the binding dynamics that are outlined in Table 1 [8,9,10]. Typically, metal binds at the cleft between two conserved domains that are connected by an α-helical linker, which closes in most SBPs to bury the metal ion. Clusters A, C, D, and H contain SBPs that bind zinc, manganese, iron, and nickel specifically. Metal binding sites within these SBPs also typically contain histidine, cysteine, aspartate, and/or glutamate residues due to the attraction between their electron-pair donors (oxygen, nitrogen, and sulfur) and metal ions [11]. Other metal transport mechanisms that have been characterized in one or more Streptococci include natural resistance-associated macrophage proteins (NRAMP); ZIP-family proteins; P-type ATPases; and cation diffusion facilitator (CDF) proteins [4,12,13]. The identification of all known systems in human-associated *Streptococcus* spp. and their respective substrates are outlined in Table 2 and discussed in the following sections.

## 2. Zinc

In bacteria, zinc (Zn) is the native metal cofactor for approximately 5% of all proteins and is involved in essential processes such as cell growth and metabolism [88]. Zn is also a highly competitive metal ion, as described by its high position on the Irving-Williams series on the stability of metal complexes [89]. Due to these binding properties, Zn is a frequent cause of mismetallation in proteins that require other metal ions to function properly, and due to its importance in cellular physiology, it is an effective target for host defenses such as nutritional immunity, where metals are sequestered to inhibit pathogen growth [5,90]. Maintaining Zn homeostasis is, therefore, essential for *Streptococcus* spp. to avoid Zn toxicity and starvation. 

### 2.1. Transport

Zn transport into bacterial cells is mainly mediated by ABC-type transport systems, while the efflux of Zn out of the cells is primarily performed by CDF pumps [88]. The high-affinity Zn transport system in Streptococci is known as AdcBC, with one or more cognate substrate binding proteins (SBPs) known as AdcA, AdcAII, and Lmb (laminin-binding protein) or Lsp (lipoprotein of *S. pyogenes*). Of note, *Streptococcus* spp. encode different combinations of Zn SBPs such as AdcA in *S. mutans*; AdcA and AdcAII in *S. pneumoniae*; AdcA and Lsp in *S. pyogenes*; and all three proteins, AdcA, AdcAII, and Lmb, in *S. agalactiae* [2,15,56]. Another unique attribute of the Streptococci is that most strains contain poly-histidine triad proteins (Pht), which are known to bind Zn and facilitate Zn uptake by interacting with SBPs [91]. The defining feature of the Pht proteins is the histidine triad motifs, HxxHxH, which are also Zn binding sites. *S. pneumoniae* encodes four Pht genes across two different operons (PhtAB and PhtDE), while other species encode fewer, such as ShtI and ShtII in *S. agalactiae* [14]. *S. mutans* is the only species of interest that has not been shown to express Pht proteins.

The most well characterized Zn efflux system is CzcD, which has been described in a few pathogenic species including *S. agalactiae*, *S. pneumoniae*, and *S. pyogenes* [16,58,88]. Recently, a unique P-type ATPase exporter named ZccE was also discovered in *S. mutans*, which, notably, does not have a CzcD homolog [30]. ZccE is shown to contribute to Zn tolerance and virulence, which is discussed in a later section.

### 2.2. Role in Virulence

Zn availability in the host during colonization and infection fluctuates based on several different factors such as cell type, tissue, and stage of infection [90,92,93,94]. Zn is also tightly regulated in the host by metal chelators such as neutrophil-derived calprotectin (CP, S100A8 and S100A9) and calgranulin C (S100A12), and by more than 20 metal transport systems belonging to the ZnT (export) and ZIP (import) family proteins [94,95]. Therefore, Streptococci encounter not only Zn limitation but also Zn toxicity during colonization and throughout the course of infection. 

#### 2.2.1. Zn Limitation

CP is one of the most well-studied metal chelators at the host–pathogen interface as it is involved in a process called nutritional immunity where pathogens are starved of available nutrients such as metal ions. CP is abundantly present at sites of infection and is mainly produced by myeloid cells, especially neutrophils where it makes up about 50% of the cytoplasmic protein content. The mechanisms by which *Streptococcus* spp. respond to and withstand CP-mediated Zn starvation in vitro and in vivo is beginning to be understood in species such as *S. pyogenes*, *S. agalactiae*, and *S. pneumoniae* [2,58,96,97,98,99]. When Streptococci are in Zn limiting conditions, it has been repeatedly found that the Adc operon, additional SBPs, and Pht proteins are necessary to maintain optimal intracellular Zn levels. The role of Zn transport in virulence is clear as Adc mutants in a variety of Streptococcal species have attenuated disease severity and/or colonization in experimental animal models such as rat tooth colonization, nasopharynx infection, meningitis, and skin infection [2,28,40,59,100,101]. The regulator AdcR has also been shown to play a role in virulence as the negative regulator of the Zn import system, as well as regulating other factors such as capsule biosynthesis in *S. pyogenes* [57,78,88,90,102]. It was recently speculated that Zn sequestration by CP may also have a beneficial effect on pathogenic bacteria by preventing the irreversible binding of Zn^2+^ to the SBP of the manganese transport system, which then inhibits manganese uptake in *S. pneumoniae* [103]. This observation remains to be seen in other Streptococci but could impact colonization and disease. 

Another strategy that Streptococci use to overcome Zn limitation is to store Zn ions [57]. The Pht surface proteins can not only pass Zn ions to the AdcA/Lmb SBPs for transport, but they can also bind up to five Zn atoms per molecule. *Streptococcal* mutants lacking Pht proteins have been shown to have decreased intracellular Zn levels, decreased resistance to Zn limitation, and decreased virulence [14,41,104]. Lastly, it was found that *S. pyogenes* replaced its abundant Zn-containing proteins with Zn-free paralogs such as the 30S ribosomal protein S14 during Zn limitation, as a mechanism to recycle the ions that were already inside the cell [57]. This phenomenon contributes to virulence in Zn limiting conditions but remains to be examined in other Streptococci.

Zn, Zn transport systems, and Pht proteins are also known to contribute to Streptococcal adherence, invasion, and biofilm formation [26,30,88,100,105,106,107]. For example, *S. agalactiae* and *S. pyogenes* AdcAII/Lmb/Lsp homologues can bind to the extracellular matrix protein laminin, which is important for adhesion and invasion into epithelial cells [107,108,109]. It is important to note that this phenotype is not consistent, as later studies found that there was no interaction with laminin in *S. agalactiae* and *S. pneumoniae* [15,100]. The Pht proteins of *S. pneumoniae* can also contribute to the adhesion to respiratory epithelial cells, as an anti-PhtD antibody was able to block bacterial attachment to cells [110]. 

#### 2.2.2. Zn Intoxication

During colonization and infection, Streptococci also experience Zn intoxication by the host. Specifically, in neutrophils, Zn is sequestered in lysosomes and azurophilic granules and released into phagosomes containing *S. pyogenes* [96]. Elevated Zn levels cause decreased virulence by attenuating hyaluronic acid capsule biosynthesis and by inhibiting important enzymes of glucose catabolism such as phosphofructokinase and GAPDH [58]. Furthermore, Zn at high concentrations inhibits biofilm formation in *Streptococcus* spp. and, therefore, pathogenicity. This has been shown in *S. pneumoniae*, *S. pyogenes*, *S. mutans*, and *S. suis* [28,106,111,112]. The Zn efflux systems in Streptococci are, therefore, necessary to overcome Zn toxicity by ridding the cell of extra Zn and contributing to Streptococcal infections [16,30,58,96,113,114,115].

Overall, Streptococci have multiple mechanisms to maintain Zn homeostasis in different environments, including import and export systems, Zn binding proteins, and Zn-dependent regulatory factors. However, there are still ranges of tolerance for Zn starvation and intoxication depending on the species and strain variability, which has been exemplified in recent publications [30,116]. Therefore, the role of Zn in *Streptococcus* spp. pathogenicity remains to be fully understood.

## 3. Manganese and Iron

Manganese (Mn) and iron (Fe) are also well characterized as important transition metals used by most organisms across all forms of life. Previous reviews have highlighted the observation that there are both Fe-centric and Mn-centric metabolic pathways in bacteria that have different mechanisms to maintain metal homeostasis [117]. Streptococci fall along the middle of this spectrum and have transport machinery, regulatory mechanisms, and pathogenic uses for both Mn and Fe, with some degree of overlap. The intricacies of Mn and Fe transport and their role in virulence in *Streptococcus* spp. has been previously described [4,12,117,118]. Therefore, this review aims to compare the role of these metals in different *Streptococcus* spp. and provide an update on what has been most recently discovered.

### 3.1. Transport

Mn import is facilitated by two major types of transporters that are present in Streptococci. The first system is an ABC-type transporter, commonly named MtsABC, but other homologs in Streptococci include, but are not limited to, SloABC, MntABC, and PsaABC. These transport systems have also been shown to transport Fe in some species such as *S. agalactiae*, *S. pyogenes*, and *S. sanguinis*, and even to bind cadmium in *S. pneumoniae*. The second type of Mn transporter is the NRAMP (natural resistance-associated macrophage protein) secondary active transporter, which, surprisingly, is not present in *S. pyogenes* or *S. pneumoniae* [18]. NRAMPs couple favorable energy of the passage of one molecule to power the transport of another and is usually named MntH [119]. Fe has also been shown to bind NRAMP transporters, but has relatively poor binding affinity compared to Mn, in striking contrast of their positions on the Irving-Williams series of cation binding affinity. MtsABC and NRAMP transporters are active at different stages of growth, with MtsABC more effective at a slightly higher pH, while NRAMPs are important for survival in acidic conditions [18,38]. A third, and less well-characterized, type of Mn importer is a ZIP-family transporter, TmpA (transporter of metal protein A), that was recently discovered in *S. sanguinis* [13]. The presence of this type of transporter and its role in metal homeostasis in other Streptococci remains to be elucidated.

There are five different classes of Mn exporters known in bacteria, including MntP type, CDF pumps, TerC type, P-type ATPases, and MneA type, but only two types have been studied in Streptococci [117]. These exporters are the CDF pump and the P-type ATPase named MntE and MgtA, respectively. MntE is commonly encoded across the Streptococci but has not yet been described in most species. Interestingly, *S. pneumoniae* is unique in that it has both types of Mn exporters; however, this could change as more Mn exporters are characterized in *Streptococcus* spp. [45,46]. For Fe, there is only one P-type ATPase efflux system, PmtA, that has been characterized in *S. pyogenes* and *S. suis* [64,85].

Fe is also imported through ABC-type transport systems but, unlike Mn, Fe can be taken up in multiple forms such as heme and siderophores. In addition, there is usually more than one Fe-dependent ABC system encoded by each species. The cellular uptake of Fe can also be in the form of ferrous Fe (Fe^2+^) or ferric Fe (Fe^3+^), with Fe^3+^ usually being the most common in microbial habitats of host organisms due to enzymatic oxidation or reactions with oxygen [12]. The most abundant source of Fe in host species is heme, and the most pathogenic Streptococci contain heme-binding ABC transporters [12,118]. In addition, chelated Fe can be acquired using hydroxamate and catecholate-type siderophores such as FhuD in *S. agalactiae*, PiuA in *S. pneumoniae*, and the unique EqbA in *S. equi* [20,24,51]. However, not all Streptococci that have siderophore transporters can produce and secrete their own, including *S. pneumoniae* and *S. pyogenes*. This suggests that they may have uptake machinery to utilize siderophores produced by other bacteria, as was previously shown in *S. pyogenes* [12]. Interestingly, many oral *Streptococcus* spp., such as *S. mutans* and *S. gordonii*, have not been shown to contain siderophore-mediated Fe uptake machinery at all [120].

### 3.2. Role in Virulence

Mn is a cofactor for many of the proteins that are involved in Streptococcal growth, replication, virulence, and biofilm formation [117]. More specifically, it is most associated with defense against reactive oxygen species (ROS), nucleotide synthesis, and normal cell physiology and development [113,121,122]. A few Mn-dependent enzymes include superoxide dismutase (SOD), ribonucleotide reductase, and Mn-dependent phosphatases [122,123,124]. A specific Mn-dependent enzyme called phosphoglucomutase (Pgm) is involved in capsular polysaccharide production and is influenced by not only Mn, but also Zn concentrations [125]. An example of this was shown in *S. pneumoniae*, where a low Mn/high Zn ratio resulted in inactive Pgm and a thinner capsule [126]. Fe is used by most organisms for a wide range of metabolic and informational cellular pathways including electron transport, peroxide reduction, and amino acid synthesis. In fact, it is estimated that bacteria require at least 10^−8^ mol/L of Fe for growth and that there are over 100 metabolic enzymes alone that require Fe [12]. The host also needs Mn and Fe and employs mechanisms to starve pathogens. As was mentioned above, CP is a known neutrophil-derived metal chelator that can sequester Mn and Fe in addition to Zn during infection. Neutrophils also release Fe specific chelators such as Lipocalin-2, which binds bacterial siderophores, and Lactoferrin, which binds Fe^3+^ directly [127,128]. To withstand host-mediated Mn and Fe starvation and to survive in metal limiting environments, Streptococci utilize their import systems, as is shown by the decreased bacterial burden and disease severity in animals that are infected with bacterial mutants lacking Mn or Fe transporters [32,43,47,48,61,76,129,130]. At the other extreme, Mn and Fe at high concentrations are also toxic to cells. Fortunately, some pathogenic Streptococci contain Mn exporters such as MntE in *S. pneumoniae*, *S. pyogenes*, and *S. suis*, and the Fe exporter PmtA in *S. pyogenes* that have been found to be critical for virulence [46,63,82,131].

Pathogenic bacteria are exposed to ROS from environmental redox reactions, intracellular enzyme autoxidation, or, most notably, from the host or competing bacteria during colonization and infection [38,117,132,133,134]. ROS are toxic to cells due to the high reactivity of Fe^2+^ with hydrogen peroxide (H_2_O_2_), resulting in hydroxyl radicals; however, Mn does not cause Fenton reactions and is often the basis for oxidative stress response in bacteria, either as a cofactor or as a nonenzymatic antioxidant [135]. Notably, the main defense system in *Streptococcus* spp. to combat oxidative stress is the enzyme SOD, and it is mainly known to use Mn^2+^ as a cofactor in Streptococci [4]. To protect the cell, SOD converts superoxide into oxygen and H_2_O_2_, which is then further broken down into water and oxygen [132]. The effect of Mn homeostasis on SOD activity and oxidative stress resistance is exemplified by the fact that Mn and Fe transporter mutants have an increased susceptibility to H_2_O_2_ and paraquat and less tolerance for low pH [32,33,43,63,122,130,136]. In addition, it was shown that cadmium ions could disrupt Mn uptake and efflux systems, which lowered intracellular concentrations and, therefore, indirectly increased *S. pneumoniae* susceptibility to oxidative stress [137]. Another conserved mechanism in Gram-positives to withstand oxidative stress is to sequester free Fe ions using Dps-like peroxide resistance proteins (Dpr), which has been shown so far in *S. pneumoniae*, *S. pyogenes*, *S. mutans*, and *S. suis* [138,139,140,141,142]. Additionally, *S. pyogenes* and *S. suis* can remove reactive Fe from the cell using their PmtA efflux system [64,85,131]. Overall, metal starvation, toxicity, and oxidative stress are common problems that pathogenic Streptococci encounter in different environments, and they encode a myriad of Mn and Fe-dependent systems and enzymes to help overcome these pressures.

## 4. Copper

Copper (Cu) is present in the human body but is only found in trace amounts compared to other transition metals. Cu has a few known roles that influence the pathogenicity of bacteria, though these are not well characterized across the Streptococci. Cu also sits atop the Irving-Williams series, surpassing Zn for binding affinity, and therefore has a very high potential to cause mismetallation, as is exemplified in *S. pneumoniae* [143]. Similarly to Fe, Cu is also redox active and can generate ROS via a Fenton-like reaction, making it toxic in high quantities [6,144]. Therefore, the control of intracellular Cu is imperative for both host and pathogen.

### 4.1. Transport

The primary bacterial systems that are characterized in the transport of Cu are involved in Cu export to prevent toxicity. In Streptococci, the only characterized Cu transport system is CopYAZ, which consists of a P-type ATPase (CopA), a cytoplasmic metallochaperone (CopZ), and the repressor (CopY). CopA couples Cu^+^ transport to ATP hydrolysis for active transport across the plasma membrane, while CopZ is involved in cytoplasmic trafficking and the shuttling of Cu ions to CopA and the regulator CopY [21,27,72,144,145,146]. A unique Cu chaperone (CupA) was discovered in *S. pneumoniae* that is only present in a few other *Streptococcus* spp., and it has been shown to reduce Cu ions from Cu^2+^ to Cu^+^, the exported Cu state, using a cupredoxin fold [147]. Aside from CopYAZ, intracellular glutathione can also bind Cu ions to aid in Cu tolerance when the exporter is overwhelmed, as was shown in *S. pyogenes* [148].

### 4.2. Role in Virulence

Cuproenzymes, or proteins that permanently bind Cu, are primarily involved in aerobic and anaerobic electron transfer reactions and superoxide dismutation, which are important for bacterial survival [144]. However, Cu at high concentrations is very toxic and the host exerts Cu stress on bacteria through pumping Cu and other metal ions into the phagosomes of macrophages to induce mismetallation, oxidative stress, and death. To deal with this pressure, several pathogenic *Streptococcus* spp. have evolved Cu efflux systems such as *S. agalactiae*, *S. pneumoniae*, and *S. pyogenes* [21,27,37,54,72,86,147,149]. These transporters are negatively regulated by CopY, which are derepressed in the presence of Cu ions. Knock-out strains lacking Cop proteins have been shown to be more sensitive to Cu intoxication, as the bacteria are unable to regulate intracellular Cu levels. Further, high Cu concentrations inhibit biofilm formation, and the detachment of *S. pyogenes*, *S. mutans*, *S. gordonii*, and a *copA* knock-out in *S. agalactiae* decreased virulence in a mouse model of systemic infection [21,27,37,72,150]. Bacteria also have mechanisms to survive Cu stress that do not involve transport, such as sequestering Cu ions with Cu-binding proteins or oxidizing Cu^+^ to the less toxic form of Cu^2+^ [146]. It was recently published that *S. agalactiae* has several genes, in addition to the cop operon, to manage Cu homeostasis during Cu stress, and these genes were predicted to be involved in cell wall biogenesis, metabolism, and signal transduction, including *oafA*, *hisMJP*, and *stp1*, respectively [151].

## 5. Nickel

Nickel (Ni) is another transition metal ion that is found in trace amounts within the human host. In fact, Ni is found at less than 5 ppm in most human organs, and mammals do not synthesize any known Ni-dependent enzymes. This suggests that Ni could be available for use by bacterial commensals or pathogens [152]. However, to date, very little is known on the role of Ni in bacterial pathogenesis.

### 5.1. Transport

Ni transport in Streptococci is largely uncharacterized, with the only known system being the ABC-type transporter UreMQO in *S. salivarius* [73]. UreMQO is encoded within the same operon as a Ni-dependent urease. However, homologs of other known ABC transport systems such as NikABCDE in *E. coli* have been found in some *Streptococcus* spp. such as *S. agalactiae* [2,130]. These streptococcal homologs have yet to be characterized but consist of a substrate binding protein (NikA), two membrane-spanning permeases (NikBC), and two ATPases (NikDE), and they were found to contribute to in vitro CP resistance in *S. agalactiae* [2]. Other types of secondary Ni transporter that are present in prokaryotes are the Ni/Cobalt transporters (NiCoT), frequently called NixA, which also have yet to be characterized in Streptococci [152].

### 5.2. Role in Virulence

The bioavailability of Ni in the human host is limited, but since mammals do not synthesize any known Ni-requiring proteins, it is possible that Ni is more available for bacteria to use to cofactor their own enzymes [152]. There are nine known enzymes in bacteria that have been shown to require Ni with varying roles in virulence: Ni-superoxide dismutase; Ni-glyoxalase; Ni-hydroxyacid racemases; Ni-acireductone dioxygenase; [NiFe] hydrogenases; urease; methyl-coenzyme M reductase; acetyl-coenzyme A decarbonylase/synthase; and carbon monoxide dehydrogenase [152,153]. Most notable of these enzymes to be involved in pathogen virulence is urease, but most *Streptococcus* spp. have not been shown to contain urease biosynthesis genes. One exception, as mentioned above, is the urease and Ni transporter system that is encoded by *S. salivarius* [73]. Aside from this, there is only one known homolog of LarA (Ni-lactate racemase) found in *S. pneumoniae* [125,154] but, collectively, the role of Ni in the pathogenesis of Streptococci remains to be elucidated.

## 6. Conclusions and Future Directions

**Figure 1:**
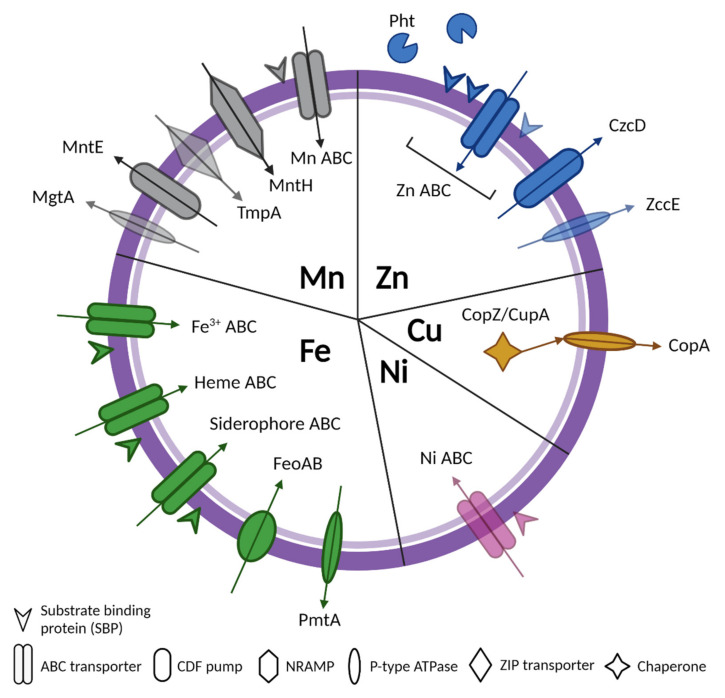
A summary diagram of all known types of transporters for Zn, Mn, Fe, Cu, and Ni in Streptococci with arrows indicating metal import or export. Transparent shapes represent transport systems that are only characterized in one species, including MgtA (*S. pneumoniae*); TmpA (*S. sanguinis*); a third zinc SBP (*S. agalactiae*); ZccE (*S. mutants*); and Ni ABC (*S. salivarius*). Created using BioRender.com.

Pathogenic Streptococci notoriously contain an arsenal of virulence factors, of which metal transport systems are critical components. This review highlights the current knowledge on Zn, Mn, Fe, Cu, and Ni transport systems that are present in *Streptococcus* spp., and their role in virulence (Figure 1). Research characterizing metal transport and regulation is still ongoing, and as most metals are used as enzymatic cofactors, the effects of metals on Streptococcal metabolism is a new and exciting research pursuit [49,114,122,125,136,155]. Metals and the mechanisms that are necessary to acquire them may also yield new drug or vaccine targets, or yield new therapies to fight infection and disease. Some examples that have been proposed or developed thus far include targeting the SBPs of transport machinery and exploiting the toxicity of metals such as silver and copper in the form of nanoparticles and metal coatings [156,157,158,159]. Though recent strides have been made in characterizing Streptococcal metal homeostasis, several important questions remain, including understanding (1) how metal availability differs across biological niches during colonization and infection; (2) what and how host factors impact Streptococcal metal homeostasis; (3) why some species rely on multiple SBPs or seemingly redundant transport systems to maintain homeostasis, while others do not; (4) how downstream Streptococcal physiology is impacted by metal starvation and/or toxicity. To this end, deciphering these mechanisms is vital to understanding how Streptococci have evolved to thwart metal stresses at the host–pathogen interface, as well as how they promote colonization and disease pathogenesis.

## Figures and Tables

**Table 1:** Summary of substrate binding protein clusters.

Cluster	Types of Ligands	Average Size (kDa)	Binding Dynamics
A	Zinc, manganese, iron, heme, siderophores	29–37	Spring hammer
B	Carbohydrates, Leu, Lle, Val, Autoinducer-2, natriuretic peptide	31–50	One domain movement
C	Di- and oligopeptides, nickel, arginine, cellobiose	59–70	One or two domain movement
D	Iron, carbohydrates, putrescine, thiamine, tetrahedral oxyanions	26–47	One domain movement
E	Sialic acid, 2-keto acids, ectoine, pyroglutamic acid	35–41	One sub-domain movement
F	Trigonal planar anions, methionine, compatible solutes, amino acids	24–60	One domain movement
G	Alginate	60	One domain movement
H	Iron	80	One sub-domain movement

**Table 2:** Characterized metal transport systems in pathogenic *Streptococcus* spp. organized by species and metal.

Organism	Substrate	Transporter/Protein	Function	References
*S. agalactiae*	Zn	Sht, ShtII/Blr	Histidine triad proteins	[14,15,16]
		AdcABC, AdcAII, Lmb	ABC transporter (import)	
		CzcD	CDF pump (export)	
	Mn, Fe	MtsABC	ABC transporter (import)	[17,18]
		MntH	NRAMP transporter (import)	
	Heme	PefAB, PefCD	ABC transporter (import)	[19]
	Siderophore	FhuCDBG	ABC transporter (import)	[20]
	Cu	CopA	P-type ATPase (export)	[21]
		CopZ	Chaperone	
*S. equi*	Heme	SeShp	Cell surface protein	[22,23]
		SeShr	Cell surface receptor	
		SeHtsABC	ABC transporter (import)	
	Siderophore	EqbHIJ	ABC transporter (import)	[24]
*S. gordonii*	Mn	ScaABC	ABC transporter (import)	[25,26]
		AdcABC	ABC transporter (import)	
	Cu	CopA	P-type ATPase (export)	[27]
		CopZ	Chaperone	
*S. mutans*	Zn	AdcABC	ABC transporter (import)	[28,29,30]
		ZccE	P-type ATPase (export)	
	Mn	MntE	CDF pump (export)	[31]
	Mn, Fe	SloABC	ABC transporter (import)	[32,33]
		MntH	NRAMP transporter (import)	
	Fe	FimA	ABC transporter element (import)	[34,35,36]
		FeoABC	Ferrous iron transport (import)	
		Smu995–998	ABC transporter (import)	
	Cu	CopA	P-type ATPase (export)	[37]
		CopZ	Chaperone	
*S. oligofermentans*	Mn	MntABC	ABC transporter (import)	[38]
		MntH	NRAMP transporter (import)	
*S. parasanguinis*	Mn, Fe	FimABC	ABC transporter (import)	[39]
*S. pneumoniae*	Zn	AdcABC AdcAII/Lmb	ABC transporter (import)	[40,41,42]
		PhtABDE	Histidine triad proteins	
		CzcD	CDF pump (export)	
	Mn, Cd, Zn	PsaABC	ABC transporter (import)	[43,44]
	Mn	MgtA	P-type ATPase (export)	[45,46]
		MntE	CDF pump (export)	
	Fe	PitABCD	ABC transporter (import)	[47]
	Hemin	SPD_1590	Hemin transporter (import)	[48,49,50]
	Siderophore	Pia/FhuDBGC	ABC transporter (import)	[48,51,52]
		PiuABCD	ABC transporter (import)	
	Cu	CopA	P-type ATPase (export)	[53,54]
		CupA	Chaperone	
*S. pyogenes*	Zn	AdcABC, AdcAII/Lmb/Lsp	ABC transporter (import)	[55,56,57,58,59]
		PhtD/HtpA, PhtY/Slr	Histidine triad proteins	
		CzcD	CDF pump (export)	
	Mn, Fe, Zn	MtsABC	ABC transporter (import)	[60,61,62]
	Mn	MntE	CDF pump (export)	[63]
	Fe	PmtA	P-type ATPase (export)	[64]
	Heme	SiuADBG/Spy383–386	ABC transporter (import)	[65,66,67,68,69,70]
		SiaABC/HtsABC	ABC transporter (import)	
		Shp	Cell surface protein	
		Shr	Cell surface receptor	
	Siderophore	FtsABCD	ABC transporter (import)	[71]
	Cu	CopA	P-type ATPase (export)	[72]
		CupA	Chaperone	
*S. salivarius*	Ni	UreMQO	ABC transporter (import)	[73]
*S. sanguinis*	Zn	SSA_0136–137, 260–261	ABC transporter (import)	[74]
	Mn	TmpA	ZIP transporter (import)	[13]
	Mn, Fe	SsaABC	ABC transporter (import)	[75,76]
		MntH	NRAMP transporter (import)	
*S. suis*	Zn	AdcABC, AdcAII	ABC transporter (import)	[77,78,79,80]
		Pht309/HtpsABC	Histidine triad protein	
	Mn	TroABCD	ABC transporter (import)	[81,82,83]
		MntE	CDF pump (export)	
	Fe	FeoAB	Ferrous iron transport (import)	[84]
	Fe, Co	PmtA	CDF pump (export)	[85]
	Cu	CopA	P-type ATPase (export)	[86]
		CopZ	Chaperone	
*S. uberis*	Mn	MtuABC	ABC transporter (import)	[87]

## References

[1] Waldron K.J., Robinson N.J. (2009). How do bacterial cells ensure that metalloproteins get the correct metal?. Nat. Rev. Microbiol..

[2] Burcham L.R., Le Breton Y., Radin J.N., Spencer B.L., Deng L., Hiron A., Ransom M.R., Mendonça J.D.C., Belew A.T., El-Sayed N.M. (2020). Identification of Zinc-Dependent Mechanisms Used by Group B Streptococcus to Overcome Calprotectin-Mediated Stress. mBio.

[3] Eom H., Song W.J. (2019). Emergence of metal selectivity and promiscuity in metalloenzymes. JBIC J. Biol. Inorg. Chem..

[4] Eijkelkamp B., McDevitt C.A., Kitten T. (2015). Manganese uptake and streptococcal virulence. BioMetals.

[5] Hood M.I., Skaar E.P. (2013). Nutritional immunity: Transition metals at the pathogen-host interface. Nat. Rev. Microbiol..

[6] Maret W. (2016). The Metals in the Biological Periodic System of the Elements: Concepts and Conjectures. Int. J. Mol. Sci..

[7] Davidson A.L., Chen J. (2004). ATP-Binding Cassette Transporters in Bacteria. Annu. Rev. Biochem..

[8] Kovacs-Simon A., Titball R.W., Michell S.L. (2011). Lipoproteins of Bacterial Pathogens. Infect. Immun..

[9] Berntsson R.P.-A., Smits S.H., Schmitt L., Slotboom D.-J., Poolman B. (2010). A structural classification of substrate-binding proteins. FEBS Lett..

[10] Chandravanshi M., Tripathi S.K., Kanaujia S.P. (2021). An updated classification and mechanistic insights into ligand binding of the substrate-binding proteins. FEBS Lett..

[11] Yamashita M.M., Wesson L., Eisenman G., Eisenberg D. (1990). Where metal ions bind in proteins. Proc. Natl. Acad. Sci. USA.

[12] Ge R., Sun X., He Q.-Y. (2009). Iron acquisition by Streptococcus species: An updated review. Front. Biol. China.

[13] Puccio T., Kunka K.S., An S., Kitten T. (2021). Contribution of a ZIP-family protein to manganese uptake and infective endocarditis virulence in *Streptococcus sanguinis*. Mol. Microbiol..

[14] Moulin P., Rong V., E Silva A.R., Pederick V.G., Camiade E., Mereghetti L., McDevitt C.A., Hiron A. (2019). Defining the Role of the *Streptococcus agalactiae* Sht-Family Proteins in Zinc Acquisition and Complement Evasion. J. Bacteriol..

[15] Moulin P., Patron K., Cano C., Zorgani M.A., Camiade E., Borezée-Durant E., Rosenau A., Mereghetti L., Hiron A. (2016). The Adc/Lmb System Mediates Zinc Acquisition in *Streptococcus agalactiae* and Contributes to Bacterial Growth and Survival. J. Bacteriol..

[16] Sullivan M.J., Goh K.G.K., Ulett G.C. (2021). Cellular Management of Zinc in Group B Streptococcus Supports Bacterial Resistance against Metal Intoxication and Promotes Disseminated Infection. mSphere.

[17] Bray B.A., Sutcliffe I.C., Harrington D.J. (2009). Expression of the MtsA lipoprotein of *Streptococcus agalactiae* A909 is regulated by manganese and iron. Antonie Van Leeuwenhoek.

[18] Shabayek S., Bauer R., Mauerer S., Mizaikoff B., Spellerberg B. (2016). A streptococcal NRAMP homologue is crucial for the survival of *Streptococcus agalactiae* under low pH conditions. Mol. Microbiol..

[19] Fernandez A., Lechardeur D., Derré-Bobillot A., Couvé E., Gaudu P., Gruss A. (2010). Two Coregulated Efflux Transporters Modulate Intracellular Heme and Protoporphyrin IX Availability in *Streptococcus agalactiae*. PLoS Pathog..

[20] Clancy A., Loar J.W., Speziali C.D., Oberg M., Heinrichs D.E., Rubens C.E. (2006). Evidence for siderophore-dependent iron acquisition in group B streptococcus. Mol. Microbiol..

[21] Sullivan M.J., Goh K.G.K., Gosling D., Katupitiya L., Ulett G.C. (2021). Copper Intoxication in Group B Streptococcus Triggers Transcriptional Activation of the cop Operon That Contributes to Enhanced Virulence during Acute Infection. J. Bacteriol..

[22] Meehan M., Burke F.M., Macken S., Owen P. (2010). Characterization of the haem-uptake system of the equine pathogen *Streptococcus equi* subsp. *equi*. Microbiology.

[23] Nygaard T.K., Liu M., McClure M.J., Lei B. (2006). Identification and characterization of the heme-binding proteins SeShp and SeHtsA of *Streptococcus equi* subspecies *equi*. BMC Microbiol..

[24] Heather Z., Holden M.T.G., Steward K.F., Parkhill J., Song L., Challis G.L., Robinson C., Davis-Poynter N., Waller A.S. (2008). A novel streptococcal integrative conjugative element involved in iron acquisition. Mol. Microbiol..

[25] Kolenbrander P.E., Andersen R.N., Baker R.A., Jenkinson H.F. (1998). The Adhesion-Associated sca Operon in *Streptococcus gordonii* Encodes an Inducible High-Affinity ABC Transporter for Mn^2+^ Uptake. J. Bacteriol..

[26] Loo C.Y., Mitrakul K., Voss I.B., Hughes C.V., Ganeshkumar N. (2003). Involvement of the adc Operon and Manganese Homeostasis in *Streptococcus gordonii* Biofilm Formation. J. Bacteriol..

[27] Mitrakul K., Loo C.Y., Hughes C.V., Ganeshkumar N. (2004). Role of a *Streptococcus gordonii* copper-transport operon, copYAZ, in biofilm detachment. Oral Microbiol. Immunol..

[28] Ganguly T., Peterson A., Kajfasz J., Abranches J., Lemos J. (2021). Zinc import mediated by AdcABC is critical for colonization of the dental biofilm by *Streptococcus mutans* in an animal model. Mol. Oral Microbiol..

[29] Pan Y., Chen Y., Chen J., Ma Q., Gong T., Yu S., Zhang Q., Zou J., Li Y. (2021). The Adc regulon mediates zinc homeostasis in *Streptococcus mutans*. Mol. Oral Microbiol..

[30] Ganguly T., Peterson A., Burkholder M., Kajfasz J.K., Abranches J., Lemos J.A. (2022). ZccE is a Novel P-type ATPase That Protects *Streptococcus mutans* against Zinc Intoxication. bioRxiv.

[31] O’Brien J., Pastora A., Stoner A., Spatafora G. (2020). The *S. mutans* mntE gene encodes a manganese efflux transporter. Mol. Oral Microbiol..

[32] Paik S., Brown A., Munro C.L., Cornelissen C.N., Kitten T. (2003). The sloABCR Operon of *Streptococcus mutans* Encodes an Mn and Fe Transport System Required for Endocarditis Virulence and Its Mn-Dependent Repressor. J. Bacteriol..

[33] Kajfasz J.K., Katrak C., Ganguly T., Vargas J., Wright L., Peters Z.T., Spatafora G.A., Abranches J., Lemos J.A. (2020). Manganese Uptake, Mediated by SloABC and MntH, Is Essential for the Fitness of *Streptococcus mutans*. mSphere.

[34] Spatafora G., Moore M., Landgren S., Stonehouse E., Michalek S. (2001). Expression of *Streptococcus mutans* fimA is iron-responsive and regulated by a DtxR homologue. Microbiology.

[35] Galvao L.C.D.C., Miller J.H., Kajfasz J.K., Scott-Anne K., Freires I., Franco G.C.N., Abranches J., Rosalen P.L., Lemos J.A. (2015). Transcriptional and Phenotypic Characterization of Novel Spx-Regulated Genes in *Streptococcus mutans*. PLoS ONE.

[36] Ganguly T., Kajfasz J.K., Miller J.H., Rabinowitz E., Galvao L.C.D.C., Rosalen P.L., Abranches J., Lemos J.A. (2018). Disruption of a Novel Iron Transport System Reverses Oxidative Stress Phenotypes of a dpr Mutant Strain of *Streptococcus mutans*. J. Bacteriol..

[37] Vats N., Lee S.F. (2001). Characterization of a copper-transport operon, copYAZ, from Streptococcus mutans. Microbiology.

[38] Wang X., Tong H., Dong X. (2014). PerR-Regulated Manganese Ion Uptake Contributes to Oxidative Stress Defense in an Oral Streptococcus. Appl. Environ. Microbiol..

[39] Oetjen J., Fives-Taylor P., Froeliger E.H. (2002). The Divergently Transcribed Streptococcus parasanguis Virulence-Associated fimA Operon Encoding an Mn^2+^ -Responsive Metal Transporter and pepO Encoding a Zinc Metallopeptidase Are Not Coordinately Regulated. Infect. Immun..

[40] Plumptre C.D., Eijkelkamp B.A., Morey J.R., Behr F., Couñago R.M., Ogunniyi A.D., Kobe B., O’Mara M.L., Paton J.C., McDevitt C.A. (2014). AdcA and AdcAII employ distinct zinc acquisition mechanisms and contribute additively to zinc homeostasis in *Streptococcus pneumoniae*. Mol. Microbiol..

[41] Plumptre C.D., Hughes C.E., Harvey R.M., Eijkelkamp B.A., McDevitt C.A., Paton J.C. (2014). Overlapping Functionality of the Pht Proteins in Zinc Homeostasis of *Streptococcus pneumoniae*. Infect. Immun..

[42] Kloosterman T.G., van der Kooi-Pol M.M., Bijlsma J.J.E., Kuipers O.P. (2007). The novel transcriptional regulator SczA mediates protection against Zn^2+^ stress by activation of the Zn^2+^-resistance gene czcD in *Streptococcus pneumoniae*. Mol. Microbiol..

[43] McAllister L.J., Tseng H.-J., Ogunniyi A.D., Jennings M.P., McEwan A.G., Paton J.C. (2004). Molecular analysis of the psa permease complex of *Streptococcus pneumoniae*. Mol. Microbiol..

[44] Manzetti S. (2018). Quantum chemical calculations of the active site of the solute-binding protein PsaA from *Streptococcus pneumoniae* explain electronic selectivity of metal binding. Struct. Chem..

[45] E Martin J., Le M.T., Bhattarai N., A Capdevila D., Shen J., E Winkler M., Giedroc D.P. (2019). A Mn-sensing riboswitch activates expression of a Mn^2+^/Ca^2+^ ATPase transporter in Streptococcus. Nucleic Acids Res..

[46] Rosch J.W., Gao G., Ridout G., Wang Y.-D., Tuomanen E.I. (2009). Role of the manganese efflux system mntE for signalling and pathogenesis in *Streptococcus pneumoniae*. Mol. Microbiol..

[47] Brown J.S., Gilliland S.M., Ruiz-Albert J., Holden D.W. (2002). Characterization of Pit, a *Streptococcus pneumoniae* Iron Uptake ABC Transporter. Infect. Immun..

[48] Brown J.S., Gilliland S.M., Holden D.W. (2001). A *Streptococcus pneumoniae* pathogenicity island encoding an ABC transporter involved in iron uptake and virulence. Mol. Microbiol..

[49] Miao X., He J., Zhang L., Zhao X., Ge R., He Q.-Y., Sun X. (2018). A Novel Iron Transporter SPD_1590 in *Streptococcus pneumoniae* Contributing to Bacterial Virulence Properties. Front. Microbiol..

[50] Tai S.S., Yu C., Lee J.K. (2003). A solute binding protein of *Streptococcus pneumoniae* iron transport. FEMS Microbiol. Lett..

[51] Zhang Y., Edmonds K.A., Raines D.J., Murphy B.A., Wu H., Guo C., Nolan E.M., VanNieuwenhze M.S., Duhme-Klair A.-K., Giedroc D.P. (2020). The pneumococcal iron uptake protein A (PiuA) specifically recognizes tetradentate FeIII bis- and mono-catechole complexes. J. Mol. Biol..

[52] Pramanik A., Braun V. (2006). Albomycin Uptake via a Ferric Hydroxamate Transport System of *Streptococcus pneumoniae* R6. J. Bacteriol..

[53] Shafeeq S., Yesilkaya H., Kloosterman T.G., Narayanan G., Wandel M., Andrew P.W., Kuipers O.P., Morrissey J.A. (2011). The cop operon is required for copper homeostasis and contributes to virulence in *Streptococcus pneumoniae*. Mol. Microbiol..

[54] Fu Y., Tsui H.-C.T., Bruce K.E., Sham L.T., Higgins K.A., Lisher J.P., Kazmierczak K.M., Maroney M.J., Dann C.E., Winkler M.E. (2013). A new structural paradigm in copper resistance in *Streptococcus pneumoniae*. Nat. Chem. Biol..

[55] Brenot A., Weston B.F., Caparon M.G. (2007). A PerR-regulated metal transporter (PmtA) is an interface between oxidative stress and metal homeostasis in *Streptococcus pyogenes*. Mol. Microbiol..

[56] Tedde V., Rosini R., Galeotti C.L. (2016). Zn^2+^ Uptake in *Streptococcus pyogenes*: Characterization of adcA and lmb Null Mutants. PLoS ONE.

[57] Sanson M., Makthal N., Flores A., Olsen R.J., Musser J.M., Kumaraswami M. (2015). Adhesin competence repressor (AdcR) from *Streptococcus pyogenes* controls adaptive responses to zinc limitation and contributes to virulence. Nucleic Acids Res..

[58] Ong C.-L., Gillen C.M., Barnett T., Walker M., McEwan A.G. (2014). An Antimicrobial Role for Zinc in Innate Immune Defense Against Group A Streptococcus. J. Infect. Dis..

[59] Weston B.F., Brenot A., Caparon M.G. (2009). The Metal Homeostasis Protein, Lsp, of *Streptococcus pyogenes* Is Necessary for Acquisition of Zinc and Virulence. Infect. Immun..

[60] Janulczyk R., Pallon J., Bjorck L. (1999). Identification and characterization of a *Streptococcus pyogenes* ABC transporter with multiple specificity for metal cations. Mol. Microbiol..

[61] Janulczyk R., Ricci S., Björck L. (2003). MtsABC Is Important for Manganese and Iron Transport, Oxidative Stress Resistance, and Virulence of *Streptococcus pyogenes*. Infect. Immun..

[62] Sun X., Ge R., Chiu J.-F., Sun H., He Q.-Y. (2008). Lipoprotein MtsA of MtsABC in *Streptococcus pyogenes* primarily binds ferrous ion with bicarbonate as a synergistic anion. FEBS Lett..

[63] Turner A., Ong C.-L., Gillen C.M., Davies M.R., West N.P., McEwan A.G., Walker M.J. (2015). Manganese Homeostasis in Group A Streptococcus Is Critical for Resistance to Oxidative Stress and Virulence. mBio.

[64] Turner A.G., Ong C.-L.Y., Djoko K.Y., West N.P., Davies M.R., McEwan A.G., Walker M.J. (2017). The PerR-Regulated P 1B-4 -Type ATPase (PmtA) Acts as a Ferrous Iron Efflux Pump in *Streptococcus pyogenes*. Infect. Immun..

[65] Montañez G.E., Neely M.N., Eichenbaum Z. (2005). The streptococcal iron uptake (Siu) transporter is required for iron uptake and virulence in a zebrafish infection model. Microbiology.

[66] Bates C.S., Montañez G.E., Woods C.R., Vincent R.M., Eichenbaum Z. (2003). Identification and Characterization of a *Streptococcus pyogenes* Operon Involved in Binding of Hemoproteins and Acquisition of Iron. Infect. Immun..

[67] Lei B., Liu M., Voyich J.M., Prater C.I., Kala S.V., DeLeo F.R., Musser J.M. (2003). Identification and Characterization of HtsA, a Second Heme-Binding Protein Made by *Streptococcus pyogenes*. Infect. Immun..

[68] Sook B.R., Block D.R., Sumithran S., Montañez G.E., Rodgers K.R., Dawson J.H., Eichenbaum Z., Dixon D.W. (2008). Characterization of SiaA, a Streptococcal Heme-Binding Protein Associated with a Heme ABC Transport System. Biochemistry.

[69] Zhu H., Liu M., Lei B. (2008). The surface protein Shr of *Streptococcus pyogenes* binds heme and transfers it to the streptococcal heme-binding protein Shp. BMC Microbiol..

[70] Lei B., Smoot L.M., Menning H.M., Voyich J.M., Kala S.V., Deleo F.R., Reid S.D., Musser J.M. (2002). Identification and Characterization of a Novel Heme-Associated Cell Surface Protein Made by *Streptococcus pyogenes*. Infect. Immun..

[71] Hanks T.S., Liu M., McClure M.J., Lei B. (2005). ABC transporter FtsABCD of *Streptococcus pyogenes* mediates uptake of ferric ferrichrome. BMC Microbiol..

[72] Young C.A., Gordon L.D., Fang Z., Holder R.C., Reid S.D. (2015). Copper Tolerance and Characterization of a Copper-Responsive Operon, copYAZ, in an M1T1 Clinical Strain of *Streptococcus pyogenes*. J. Bacteriol..

[73] Chen Y.-Y.M., Burne R.A. (2003). Identification and Characterization of the Nickel Uptake System for Urease Biogenesis in Streptococcus salivarius 57.I. J. Bacteriol..

[74] Li K., Gifford A.H., Hampton T.H., O’Toole G.A. (2020). Availability of Zinc Impacts Interactions between *Streptococcus sanguinis* and *Pseudomonas aeruginosa* in Coculture. J. Bacteriol..

[75] Puccio T., An S., Schultz A.C., Lizarraga C.A., Bryant A.S., Culp D.J., Burne R.A., Kitten T. (2022). Manganese transport by *Streptococcus sanguinis* in acidic conditions and its impact on growth in vitro and in vivo. Mol. Microbiol..

[76] Crump K.E., Bainbridge B., Brusko S., Turner L.S., Ge X., Stone V., Xu P., Kitten T. (2014). The relationship of the lipoprotein SsaB, manganese and superoxide dismutase in *Streptococcus sanguinis* virulence for endocarditis. Mol. Microbiol..

[77] Aranda J., Teixidó L., Fittipaldi N., Cortés P., Llagostera M., Gottschalk M., Barbé J. (2012). Inactivation of the gene encoding zinc-binding lipoprotein 103 impairs the infectivity of *Streptococcus suis*. Can. J. Vet. Res..

[78] Zheng C., Qiu J., Zhao X., Yu S., Wang H., Wan M., Wei M., Jiao X. (2022). The AdcR-regulated AdcA and AdcAII contribute additively to zinc acquisition and virulence in *Streptococcus suis*. Vet. Microbiol..

[79] Shao Z.-Q., Pan X., Li X., Liu W., Han M., Wang C., Wang J., Zheng F., Cao M., Tang J. (2011). HtpS, a novel immunogenic cell surface-exposed protein of *Streptococcus suis*, confers protection in mice. FEMS Microbiol. Lett..

[80] Li M., Shao Z.-Q., Guo Y., Wang L., Hou T., Hu D., Zheng F., Tang J., Wang C., Feng Y. (2015). The type II histidine triad protein HtpsC is a novel adhesion with the involvement of *Streptococcus suis* virulence. Virulence.

[81] Zheng C., Wei M., Qiu J., Jia M., Zhou X., Jiao X. (2021). TroR Negatively Regulates the TroABCD System and Is Required for Resistance to Metal Toxicity and Virulence in *Streptococcus suis*. Appl. Environ. Microbiol..

[82] Xu J., Zheng C., Cao M., Zeng T., Zhao X., Shi G., Chen H., Bei W. (2017). The manganese efflux system MntE contributes to the virulence of *Streptococcus suis* serotype 2. Microb. Pathog..

[83] Schreur P.J.W., Rebel J.M.J., Smits M.A., van Putten J.P.M., Smith H.E. (2011). TroA of *Streptococcus suis* Is Required for Manganese Acquisition and Full Virulence. J. Bacteriol..

[84] Aranda J., Cortés P. (2009). Contribution of the FeoB transporter to *Streptococcus suis* virulence. Int. Microbiol..

[85] Zheng C., Jia M., Gao M., Lu T., Li L., Zhou P. (2019). PmtA functions as a ferrous iron and cobalt efflux pump in *Streptococcus suis*. Emerg. Microbes Infect..

[86] Zheng C., Jia M., Lu T., Gao M., Li L. (2019). CopA Protects *Streptococcus suis* against Copper Toxicity. Int. J. Mol. Sci..

[87] Smith A.J., Ward P.N., Field T.R., Jones C.L., Lincoln R.A., Leigh J.A. (2003). MtuA, a Lipoprotein Receptor Antigen from Streptococcus uberis, Is Responsible for Acquisition of Manganese during Growth in Milk and Is Essential for Infection of the Lactating Bovine Mammary Gland. Infect. Immun..

[88] Shafeeq S., Kuipers O.P., Kloosterman T.G. (2013). The role of zinc in the interplay between pathogenic streptococci and their hosts. Mol. Microbiol..

[89] Irving H., Williams R.J.P. (1953). 637. The stability of transition-metal complexes. J. Chem. Soc..

[90] Makthal N., Kumaraswami M. (2017). Zinc’ing it out: Zinc homeostasis mechanisms and their impact on the pathogenesis of human pathogen group A streptococcus. Metallomics.

[91] Plumptre C.D., Ogunniyi A.D., Paton J.C. (2012). Polyhistidine triad proteins of pathogenic streptococci. Trends Microbiol..

[92] Corbin W.J., Seeley B.D., Raab E.H., Feldmann A., Miller J., Torres M.R., Anderson V.J., Dattilo K.L., Anderson B.M., Dunman K.L. (2008). Bacterial Growth in Tissue Abscesses. Science.

[93] Eijkelkamp B.A., Morey J.R., Neville S.L., Tan A., Pederick V.G., Cole N., Singh P.P., Ong C.-L., De Vega R.G., Clases D. (2019). Dietary zinc and the control of *Streptococcus pneumoniae* infection. PLoS Pathog..

[94] Kehl-Fie T.E., Skaar E.P. (2010). Nutritional immunity beyond iron: A role for manganese and zinc. Curr. Opin. Chem. Biol..

[95] Lichten L.A., Cousins R.J. (2009). Mammalian Zinc Transporters: Nutritional and Physiologic Regulation. Annu. Rev. Nutr..

[96] Ong C.-L.Y., Berking O., Walker M., McEwan A.G. (2018). New Insights into the Role of Zinc Acquisition and Zinc Tolerance in Group A Streptococcal Infection. Infect. Immun..

[97] Makthal N., Nguyen K., Do H., Gavagan M., Chandrangsu P., Helmann J.D., Olsen R.J., Kumaraswami M. (2017). A Critical Role of Zinc Importer AdcABC in Group A Streptococcus-Host Interactions during Infection and Its Implications for Vaccine Development. eBioMedicine.

[98] De Filippo K., Neill D.R., Mathies M., Bangert M., McNeill E., Kadioglu A., Hogg N. (2014). A new protective role for S100A9 in regulation of neutrophil recruitment during invasive pneumococcal pneumonia. FASEB J..

[99] Makthal N., Do H., Wendel B.M., Olsen R.J., Helmann J.D., Musser J.M., Kumaraswami M. (2020). Group A Streptococcus AdcR Regulon Participates in Bacterial Defense against Host-Mediated Zinc Sequestration and Contributes to Virulence. Infect. Immun..

[100] Brown L.R., Gunnell S.M., Cassella A.N., Keller L.E., Scherkenbach L.A., Mann B., Brown M., Hill R., Fitzkee N.C., Rosch J.W. (2016). AdcAII of *Streptococcus pneumoniae* Affects Pneumococcal Invasiveness. PLoS ONE.

[101] Bayle L., Chimalapati S., Schoehn G., Brown J., Vernet T., Durmort C. (2011). Zinc uptake by *Streptococcus pneumoniae* depends on both AdcA and AdcAII and is essential for normal bacterial morphology and virulence. Mol. Microbiol..

[102] Shafeeq S., Kloosterman T.G., Kuipers O.P. (2011). Transcriptional response of *Streptococcus pneumoniae* to Zn^2+^ limitation and the repressor/activator function of AdcR. Metallomics.

[103] Rosen T., Hadley R.C., Bozzi A.T., Ocampo D., Shearer J., Nolan E.M. (2022). Zinc sequestration by human calprotectin facilitates manganese binding to the bacterial solute-binding proteins PsaA and MntC. Metallomics.

[104] Ogunniyi A.D., Grabowicz M., Mahdi L.K., Cook J., Gordon D.L., Sadlon T.A., Paton J.C. (2009). Pneumococcal histidine triad proteins are regulated by the Zn^2+^ -dependent repressor AdcR and inhibit complement deposition through the recruitment of complement factor H. FASEB J..

[105] Song X.-M., Connor W., Hokamp K., A Babiuk L., A Potter A. (2008). *Streptococcus pneumoniae* early response genes to human lung epithelial cells. BMC Res. Notes.

[106] Brown L.R., Caulkins R.C., Schartel T.E., Rosch J.W., Honsa E.S., Schultz-Cherry S., Meliopoulos V.A., Cherry S., Thornton J.A. (2017). Increased Zinc Availability Enhances Initial Aggregation and Biofilm Formation of *Streptococcus pneumoniae*. Front. Cell. Infect. Microbiol..

[107] Terao Y., Kawabata S., Kunitomo E., Nakagawa I., Hamada S. (2002). Novel Laminin-Binding Protein of *Streptococcus pyogenes*, Lbp, Is Involved in Adhesion to Epithelial Cells. Infect. Immun..

[108] Elsner A., Kreikemeyer B., Braun-Kiewnick A., Spellerberg B., Buttaro B.A., Podbielski A. (2002). Involvement of Lsp, a Member of the LraI-Lipoprotein Family in *Streptococcus pyogenes*, in Eukaryotic Cell Adhesion and Internalization. Infect. Immun..

[109] Spellerberg B., Rozdzinski E., Martin S., Weber-Heynemann J., Schnitzler N., Lütticken R., Podbielski A. (1999). Lmb, a Protein with Similarities to the LraI Adhesin Family, Mediates Attachment of *Streptococcus agalactiae* to Human Laminin. Infect. Immun..

[110] Kallio A., Sepponen K., Hermand P., Denoël P., Godfroid F., Melin M. (2014). Role of Pht Proteins in Attachment of *Streptococcus pneumoniae* to Respiratory Epithelial Cells. Infect. Immun..

[111] Wu C., Labrie J., Tremblay Y., Haine D., Mourez M., Jacques M. (2013). Zinc as an agent for the prevention of biofilm formation by pathogenic bacteria. J. Appl. Microbiol..

[112] Danilova T.A., Danilina G.A., Adzhieva A.A., Vostrova E.I., Zhukhovitskii V.G., Cheknev S.B. (2020). Inhibitory Effect of Copper and Zinc Ions on the Growth of *Streptococcus pyogenes* and Escherichia coli Biofilms. Bull. Exp. Biol. Med..

[113] Martin J.E., Lisher J.P., Winkler M.E., Giedroc D.P. (2017). Perturbation of manganese metabolism disrupts cell division in *Streptococcus pneumoniae*. Mol. Microbiol..

[114] Brazel E.B., Tan A., Neville S.L., Iverson A.R., Udagedara S.R., Cunningham B.A., Sikanyika M., De Oliveira D.M., Keller B., Bohlmann L. (2022). Dysregulation of *Streptococcus pneumoniae* zinc homeostasis breaks ampicillin resistance in a pneumonia infection model. Cell Rep..

[115] Martin J.E., Giedroc D.P. (2016). Functional Determinants of Metal Ion Transport and Selectivity in Paralogous Cation Diffusion Facilitator Transporters CzcD and MntE in *Streptococcus pneumoniae*. J. Bacteriol..

[116] Francis J.D., Guevara M.A., Lu J., Madhi S.A., Kwatra G., Aronoff D.M., Manning S.D., Gaddy J.A. (2022). The antimicrobial activity of zinc against group B Streptococcus is strain-dependent across diverse sequence types, capsular serotypes, and invasive versus colonizing isolates. BMC Microbiol..

[117] Bosma E.F., Rau M.H., A van Gijtenbeek L., Siedler S. (2021). Regulation and distinct physiological roles of manganese in bacteria. FEMS Microbiol. Rev..

[118] Ge R., Sun X. (2014). Iron acquisition and regulation systems in Streptococcus species. Metallomics.

[119] Kehres D.G., Zaharik M.L., Finlay B.B., Maguire M.E. (2000). The NRAMP proteins of Salmonella typhimurium and Escherichia coli are selective manganese transporters involved in the response to reactive oxygen. Mol. Microbiol..

[120] Whalan R.H., Funnell S.G.P., Bowler L.D., Hudson M.J., Robinson A., Dowson C.G. (2006). Distribution and Genetic Diversity of the ABC Transporter Lipoproteins PiuA and PiaA within *Streptococcus pneumoniae* and Related Streptococci. J. Bacteriol..

[121] Kuipers K., Gallay C., Martinek V., Rohde M., Martínková M., Van Der Beek S.L., Jong W., Venselaar H., Zomer A., Bootsma H. (2016). Highly conserved nucleotide phosphatase essential for membrane lipid homeostasis in *Streptococcus pneumoniae*. Mol. Microbiol..

[122] Puccio T., Kunka K.S., Zhu B., Xu P., Kitten T. (2020). Manganese Depletion Leads to Multisystem Changes in the Transcriptome of the Opportunistic Pathogen *Streptococcus sanguinis*. Front. Microbiol..

[123] Geno K.A., Hauser J.R., Gupta K., Yother J. (2014). *Streptococcus pneumoniae* Phosphotyrosine Phosphatase CpsB and Alterations in Capsule Production Resulting from Changes in Oxygen Availability. J. Bacteriol..

[124] Hardy G.G., Magee A.D., Ventura C.L., Caimano M.J., Yother J. (2001). Essential Role for Cellular Phosphoglucomutase in Virulence of Type 3 *Streptococcus pneumoniae*. Infect. Immun..

[125] Ong C.-L.Y., Walker M.J., McEwan A.G. (2015). Zinc disrupts central carbon metabolism and capsule biosynthesis in *Streptococcus pyogenes*. Sci. Rep..

[126] McFarland A.L., Bhattarai N., Joseph M., Winkler M.E., Martin J.E. (2021). Cellular Mn/Zn ratio influences phosphoglucomutase activity and capsule production in *Streptococcus pneumoniae* D39. J. Bacteriol..

[127] Goetz D.H., Holmes M.A., Borregaard N., Bluhm M.E., Raymond K.N., Strong R.K. (2002). The Neutrophil Lipocalin NGAL Is a Bacteriostatic Agent that Interferes with Siderophore-Mediated Iron Acquisition. Mol. Cell.

[128] Kell D.B., Heyden E.L., Pretorius E. (2020). The Biology of Lactoferrin, an Iron-Binding Protein That Can Help Defend Against Viruses and Bacteria. Front. Immunol..

[129] Cao K., Zhang T., Li N., Yang X.-Y., Ding J., He Q.-Y., Sun X. (2022). Identification and Tetramer Structure of Hemin-Binding Protein SPD_0310 Linked to Iron Homeostasis and Virulence of *Streptococcus pneumoniae*. mSystems.

[130] Burcham L.R., Akbari M.S., Alhajjar N., Keogh R.A., Radin J.N., Kehl-Fie T.E., Belew A.T., El-Sayed N.M., McIver K.S., Doran K.S. (2022). Genomic Analyses Identify Manganese Homeostasis as a Driver of Group B Streptococcal Vaginal Colonization. mBio.

[131] VanderWal A.R., Makthal N., Pinochet-Barros A., Helmann J.D., Olsen R.J., Kumaraswami M. (2017). Iron Efflux by PmtA Is Critical for Oxidative Stress Resistance and Contributes Significantly to Group A Streptococcus Virulence. Infect. Immun..

[132] Imlay J.A. (2008). Cellular Defenses against Superoxide and Hydrogen Peroxide. Annu. Rev. Biochem..

[133] Nguyen G.T., Green E.R., Mecsas J. (2017). Neutrophils to the ROScue: Mechanisms of NADPH Oxidase Activation and Bacterial Resistance. Front. Cell. Infect. Microbiol..

[134] Chen Z., Wang X., Yang F., Hu Q., Tong H., Dong X. (2017). Molecular Insights into Hydrogen Peroxide-sensing Mechanism of the Metalloregulator MntR in Controlling Bacterial Resistance to Oxidative Stresses. J. Biol. Chem..

[135] Anjem A., Varghese S., Imlay J.A. (2009). Manganese import is a key element of the OxyR response to hydrogen peroxide inEscherichia coli. Mol. Microbiol..

[136] Johnston J.W., Briles D.E., Myers L.E., Hollingshead S.K. (2006). Mn^2+^ -Dependent Regulation of Multiple Genes in *Streptococcus pneumoniae* through PsaR and the Resultant Impact on Virulence. Infect. Immun..

[137] Begg S.L., Eijkelkamp B., Luo Z., Couñago R., Morey J.R., Maher M., Ong C.-L., McEwan A.G., Kobe B., O’Mara M. (2015). Dysregulation of transition metal ion homeostasis is the molecular basis for cadmium toxicity in *Streptococcus pneumoniae*. Nat. Commun..

[138] Yamamoto Y., Higuchi M., Poole L.B., Kamio Y. (2000). Role of the dpr Product in Oxygen Tolerance in *Streptococcus mutans*. J. Bacteriol..

[139] Tsou C.-C., Chiang-Ni C., Lin Y.-S., Chuang W.-J., Lin M.-T., Liu C.-C., Wu J.-J. (2008). An Iron-Binding Protein, Dpr, Decreases Hydrogen Peroxide Stress and Protects *Streptococcus pyogenes* against Multiple Stresses. Infect. Immun..

[140] Haikarainen T., Thanassoulas A., Stavros P., Nounesis G., Haataja S., Papageorgiou A.C. (2011). Structural and Thermodynamic Characterization of Metal Ion Binding in *Streptococcus suis* Dpr. J. Mol. Biol..

[141] Hua C.-Z., Howard A., Malley R., Lu Y.-J. (2014). Effect of Nonheme Iron-Containing Ferritin Dpr in the Stress Response and Virulence of Pneumococci. Infect. Immun..

[142] Kauko A., Haataja S., Pulliainen A.T., Finne J., Papageorgiou A.C. (2004). Crystal Structure of *Streptococcus suis* Dps-like Peroxide Resistance Protein Dpr: Implications for Iron Incorporation. J. Mol. Biol..

[143] Johnson M., Kehl-Fie T., Rosch J.W. (2015). Copper intoxication inhibits aerobic nucleotide synthesis in *Streptococcus pneumoniae*. Metallomics.

[144] Andrei A., Öztürk Y., Khalfaoui-Hassani B., Rauch J., Marckmann D., Trasnea P.-I., Daldal F., Koch H.-G. (2020). Cu Homeostasis in Bacteria: The Ins and Outs. Membranes.

[145] Arguello J.M., Raimunda D., Padilla-Benavides T. (2013). Mechanisms of copper homeostasis in bacteria. Front. Cell. Infect. Microbiol..

[146] Ladomersky E., Petris M.J. (2015). Copper tolerance and virulence in bacteria. Metallomics.

[147] Neubert M.J., Dahlmann E.A., Ambrose A., Johnson M.D.L. (2017). Copper Chaperone CupA and Zinc Control CopY Regulation of the Pneumococcal cop Operon. mSphere.

[148] Stewart L.J., Ong C.-L.Y., Zhang M.M., Brouwer S., McIntyre L., Davies M.R., Walker M.J., McEwan A.G., Waldron K.J., Djoko K.Y. (2020). Role of Glutathione in Buffering Excess Intracellular Copper in *Streptococcus pyogenes*. mBio.

[149] Johnson M.D.L., Kehl-Fie T.E., Klein R., Kelly J., Burnham C., Mann B., Rosch J.W. (2015). Role of Copper Efflux in Pneumococcal Pathogenesis and Resistance to Macrophage-Mediated Immune Clearance. Infect. Immun..

[150] Vats N., Lee S.F. (2000). Active detachment of *Streptococcus mutans* cells adhered to epon–hydroxylapatite surfaces coated with salivary proteins in vitro. Arch. Oral Biol..

[151] Goh K.G.K., Sullivan M.J., Ulett G.C. (2022). The Copper Resistome of Group B Streptococcus Reveals Insight into the Genetic Basis of Cellular Survival during Metal Ion Stress. J. Bacteriol..

[152] Maier R.J., Benoit S.L. (2019). Role of Nickel in Microbial Pathogenesis. Inorganics.

[153] Mulrooney S.B., Hausinger R. (2003). Nickel uptake and utilization by microorganisms. FEMS Microbiol. Rev..

[154] Desguin B., Urdiain-Arraiza J., Da Costa M., Fellner M., Hu J., Hausinger R.P., Desmet T., Hols P., Soumillion P. (2020). Uncovering a superfamily of nickel-dependent hydroxyacid racemases and epimerases. Sci. Rep..

[155] Burcham L.R., A Hill R., Caulkins R.C., Emerson J.P., Nanduri B., Rosch J.W., Fitzkee N.C., A Thornton J. (2020). *Streptococcus pneumoniae* metal homeostasis alters cellular metabolism. Metallomics.

[156] Lemire J.A., Harrison J.J., Turner R.J. (2013). Antimicrobial activity of metals: Mechanisms, molecular targets and applications. Nat. Rev. Microbiol..

[157] Counago R.M., McDevitt C.A., Ween M.P., Kobe B. (2012). Prokaryotic substrate-binding proteins as targets for antimicrobial therapies. Curr. Drug Targets.

[158] Sánchez-López E., Gomes D., Esteruelas G., Bonilla L., Lopez-Machado A.L., Galindo R., Cano A., Espina M., Ettcheto M., Camins A. (2020). Metal-Based Nanoparticles as Antimicrobial Agents: An Overview. Nanomaterials.

[159] Birkett M., Dover L., Lukose C.C., Zia A.W., Tambuwala M.M., Serrano-Aroca A. (2022). Recent Advances in Metal-Based Antimicrobial Coatings for High-Touch Surfaces. Int. J. Mol. Sci..

